# Public Preferences for Surgeon Gender in Saudi Arabia: A Cross-Sectional Analysis

**DOI:** 10.3390/healthcare12121185

**Published:** 2024-06-12

**Authors:** Nasser A. Hakami, Hafiz I. Al-Musawa, Ali I. Alharbi, Nawaf A. Marwahi, Abdulaziz S. Almutlaq, Rayan A. Alghamdi, Sadeem M. Alshammari, Abdulaziz A. Almalki, Mohammed E. Mojiri, Naif K. Mahzara, Amro A. Hakami, Abdulrhman A. Nemri

**Affiliations:** 1 General Surgery, Surgical Department, College of Medicine, Jazan University, Jazan 45142, Saudi Arabia; nahakami@jazanu.edu.sa; 2 Faculty of Medicine, Jazan University, Jazan 45141, Saudi Arabia; 3 College of Medicine, King Faisal University, Alhasa 31982, Saudi Arabia; 4 College of Medicine, Imam Mohammad Ibn Saud Islamic University, Riyadh 11623, Saudi Arabia; 5 College of Medicine, University of Hail, Hail 55476, Saudi Arabia; 6 College of Medicine, Ibn Sina National College, Jeddah 22421, Saudi Arabia; 7 Surgery Department, King Saud University Medical City, Riyadh 12372, Saudi Arabia; 8 Security Forced Hospital, Riyadh 11481, Saudi Arabia

**Keywords:** societal, preferences, genders of surgeons, Saudi Arabia

## Abstract

Background: Gender equity in healthcare ensures equal access to services and resources for all individuals, regardless of gender. Studies show that patients’ gender influences their healthcare experiences and decisions, and societal gender preferences impact surgeon selection. Therefore, the objective of this study was to address the surgeons’ gender preferences among Saudi Arabia’s population. Methods: This study used a cross-sectional research design and survey methodology to gather data from a representative sample of adults aged 18 and older from the general population in Saudi Arabia. This study used SPSS version 26 for statistical data entry and analysis, employing descriptive and inferential statistics, frequency distributions, descriptive statistics, and multivariate analysis using multiple logistic regression for variables significantly associated with population gender in univariate analysis. Results: This study involved 2085 participants, with 51.2% aged 18–25, 18.4% aged 26–35, 16.7% aged 36–45, and 13.8% aged 45 and above. The majority were Saudi Arabian, with a majority holding a university degree. The majority worked in the healthcare sector, while the remaining 29.7% were unemployed. This study found that there were significant gender preferences among survey respondents for different types of surgical procedures. Male participants preferred male surgeons for routine non-emergency visits and sensitive obstetric, genital, sexual, and minor procedures (*p* < 0.001). Female participants, on the other hand, had no preference for emergency surgeries and major surgical procedures (*p* < 0.001). This study found that participants were more likely to prefer female surgeons for regular non-emergency visits to the surgery clinic (*p* < 0.001; aOR = 2.344). Additionally, participants had a high preference for female surgeons for sensitive cases (*p* < 0.001; aOR = 7.064) and minor surgical procedures (*p* < 0.001; aOR = 2.489). Conclusion: This study underscores the significance of cognizance and the incorporation of a patient’s gender preferences when selecting surgical procedures, thus fostering an environment that is more accommodating and focused on the patient.

## 1. Introduction

Gender equity pertains to the principle of providing equal access to healthcare services and resources for all individuals, irrespective of their gender. Recent studies have begun to investigate the ways in which the gender of patients impacts their healthcare encounters and decisions, encompassing their inclinations toward particular healthcare providers [1,2]. By investigating these elements, policymakers and healthcare providers can collaborate in an effort to rectify gender-related inequalities in the healthcare sector and advance healthcare services that are more inclusive for each patient. Gender equity is an essential component of healthcare, and recent studies have investigated the impact of gender on the preferences and experiences of patients [3,4]. Some research has explored healthcare providers’ general gender preferences, but the impact of societal gender preferences on surgeon selection remains largely unexplored. They ensure that healthcare providers receive adequate support in their professional development and that patients receive the care they desire [5,6]. The issue of gender equity in healthcare has persisted, as research has indicated that patients may have predilections regarding healthcare providers of a particular gender [7,8]. Researchers’ investigations have indicated that gender preferences might be subject to the influence of numerous factors, such as personal experiences, cultural conventions, and biased beliefs. When considering surgical procedures, it is critical to comprehend the ways in which these preferences might impact the selection of surgical care by patients [9,10,11].

Several studies have demonstrated the impact of healthcare providers’ gender on patients’ preferences and experiences. In Saudi Arabia, research found that women preferred female physicians for gynecological procedures [12]. Additional research has indicated that the gender preferences of patients might differ based on the specific surgical procedure and the cultural heritage of the individual [13,14,15]. Some studies have explored the general gender preferences of physicians, but the impact of societal gender preferences on surgeon selection remains largely unexplored.

The gender of surgeons, as an essential element of the healthcare system, may impact patients’ preferences and experiences during surgical procedures. Public preferences for the gender of surgeons are largely shaped by the cultural features and traditional values prevalent in a given society. In more conservative societies, such as Saudi Arabia, these preferences can be interpreted through the lens of unique cultural and social norms. Understanding these preferences comprehensively is crucial to ensuring support for healthcare providers in their professional development and ensuring that patients receive the care they desire. Consequently, the purpose of this research was to investigate societal preferences concerning the gender of surgeons among the general public of Saudi Arabia.

## 2. Methods

### 2.1. Study Setting and Participants

This study used a cross-sectional research design and survey methodology to collect data from a representative sample of participants. A validated questionnaire was obtained from a study conducted in Saudi Arabia [16]. The questionnaire consisted of (1) sociodemographic data, (2) questions to measure the societal gender preferences of surgeons of the Saudi population, (3) preferences for breast cancer and hemorrhoid surgery and its relationship with participants’ gender, and (4) reasons for choosing surgeon’s gender for breast cancer and hemorrhoid surgery. We used convenience sampling and reached the general public through Telegram, Twitter, and WhatsApp to ensure the convenience of the sample in terms of the time and place. The goal to use these social networking platforms was predetermined by their popularity and broad and diverse membership base. These methods allowed for the distribution of the survey in a rapid and timely manner. Although convenience sampling is known to create a limitation regarding the sample’s representativeness and the generalizability of the research, we targeted numerous groups and communities on different social networking platforms to broaden the diversity of the participants. This study encompassed all adults aged 18 years and older from the general population, regardless of occupation (medical doctors, university students, high school students, university staff, and all other members of the community in the private and public sectors). Following the IRB approval from Jazan University (REC-44/11/714; 19 June 2023), the researchers posed and shared the questionnaire on various social networking platforms to gauge the preferences of the Saudi Arabian community regarding the research objectives.

### 2.2. Sample Size Calculation

The sample size was determined for this research using a formula specific to cross-sectional study designs (n = z2 P(1 − P)/d2) based on the following criteria: an anticipated population proportion (P) of 50%; a z value of 1.96 with a 95% confidence level; and d, which represents the degree of precision where the error does not exceed 5%. 

### 2.3. Data Analysis

The statistical data entry and analysis were conducted using version 26 of the Statistical Package for the Social Sciences (SPSS). The analysis employed both descriptive and inferential statistics, depending on the intended goal of each relationship. We computed descriptive statistics after obtaining frequency distributions. Certain associations were examined at a higher level of data analysis using the chi-square test, and multivariate analysis was performed using multiple logistic regression for variables significantly associated with population gender in the univariate analysis. It was tested for multicollinearity, and no multicollinearity was found.

### 2.4. Ethical Considerations

This study was conducted in compliance with Saudi Arabia’s ethical standards and the provided guidelines. The complete assent from all participants was obtained after introducing them to this study. Those who consented completed a self-administered, anonymous questionnaire that served as the basis for our data collection. The participants were allowed to withdraw at any time without causing harm or forfeiting any benefits to those who chose to continue or discontinue their involvement. The precautions were taken to safeguard the participants’ personal information and maintain strict confidentiality.

## 3. Results

Table 1 displays the sociodemographic information of the participants. Out of the 2085 participants in this study, 51.2% were between the ages of 18 and 25. The age category of 26–35 comprised 18.4% of the participants, ranking as the second largest. The age group of 36–45 years comprised 16.7% of the participants, whereas individuals aged 45 and above constituted 13.8% of the sample. With regard to the gender composition of the participants, 40.9% identified as male, whereas 59.1% identified as female. This study revealed that 57.6% of the participants identified as single, 36.9% as married, 1.9% as divorced, and 3.5% as bereaved. With regard to nationality, this study predominantly comprised individuals from Saudi Arabia, constituting 95.3% of the sample. Non-Saudi individuals constituted 4.7% of the overall sample. In relation to educational attainment, a significant proportion of the participants (70.7%) possessed a university degree, while 24.2% had completed general education (primary, intermediate, or secondary). A lesser percentage (5.1%) possessed a postgraduate degree. The participants’ employment status revealed that 26.9% were involved in healthcare-related studies or work, while 43.4% held jobs in non-healthcare-related fields. An additional 29.7% of respondents indicated that they were unemployed. The Kingdom of Saudi Arabia dispersed the participants geographically. Following 20.5% of the participants from the Central Region were 19.9% from the Northern Region, 20.0% from the Western Region, and 20.0% from the Eastern Region.

Table 2 lists the individuals’ surgical histories. A total of 51.7% of the participants reported prior consultation with a surgeon. A total of 33.3% of participants selected a surgeon of the same gender to perform a thoracic examination. Following that came the pelvis (28.3%), abdomen (29.9%), and genitals (51.5%). The back (21.6%), face (23.5%), mouth (19.1%), head (16.1%), neck (17.4%), and extremities (13.6%) were also preferable locations. A considerable proportion of people (28.5%) lacked any preferences. The participants provided a range of justifications for their preference for a surgeon of the same gender. A total of 32.6% of participants cited comfort as the primary reason. An additional 3% cited modesty, 52.4% cited the ability to engage in frank dialogue with a surgeon of the same gender; 6.1% cited the belief that a surgeon of the same gender had a superior understanding of symptoms, and 5.6% cited confusion.

Table 3 details the gender preferences of participants for hemorrhoid and breast cancer surgeons. For breast cancer surgery, the majority of participants (53.8% of males and 52% of females) had no surgeon preference. A significant proportion of respondents (28.5% females, 21.8% males) had a preference for female surgeons, whereas a smaller percentage (24.4% males, 19.5% females) had the same preference (*p* < 0.001). In general, patients who underwent hemorrhoid surgery did not specify the gender of their surgeon (46.5% males and 47.8% females). A significant proportion of the participants (8.7% males, 40.6% females) exhibited a preference for female surgeons, whereas a minority (11.7% females, 44.8% males) did not (*p* < 0.001).

Table 4 details the gender preferences of participants for hemorrhoid and breast cancer surgeons. Participants favored female surgeons for breast cancer surgery due to their exhibiting the following qualities: fearlessness (9.7%); confidence (14.3%); patience (3.4%); expertise (8.2%); competence (4.8%); and a conducive atmosphere for candid communication (45.3%). A total of 8% of participants cited fearlessness as a factor favoring male breast cancer surgeons, while 15.1% cited confidence; 6.5% cited forbearance; 22% cited experience, and 21.4% cited competence. An unfavorable encounter with surgeons of the opposite gender (1.5% for females and 2.2% for males) influenced the participants’ gender preferences. A reduced proportion of respondents indicated superior patient examination (9.1% for female surgeons vs. 3.8% for male surgeons) and enhanced professional capabilities (3.7% for female surgeons vs. 8.0% for male surgeons). A total of 12.7% of participants cited fearlessness as a reason for preferring female surgeons for hemorrhoid surgery, followed by confidence (9.4%), fortitude (2.3%), experience (4.4%), competence (4.2%), and the ability to foster an atmosphere conducive to open communication (57.1%). A total of 7.4% of participants cited fearlessness as their preference for male surgeons during hemorrhoid surgery, with confidence (10.8%), patience (4.2%), experience (11.6%), and competency (13.9%) following closely behind. Certain respondents expressed dissatisfaction with surgeons of the opposing gender (1.0% for male surgeons and 2.6% for female surgeons), enhanced patient examination (5.6% versus 4.2%), and superior professional abilities (2.8% for female surgeons and 6.8% for male surgeons).

Table 5 presents a multivariate analysis of participants’ gender preferences for surgeons’ gender. For regular non-emergency visits to the surgery clinic, participants were more likely to prefer female surgeons (*p* < 0.001; aOR = 2.344). Furthermore, participants demonstrated high preferences for female surgeons with regard to sensitive emergency cases (*p* < 0.001; aOR = 7.064) and minor surgical procedures (*p* < 0.001; aOR = 2.489).

Table 6 presents the surgical preferences of survey respondents across a range of conditions. Male and female patients at the surgery clinic exhibited variations in their routine non-emergency visits. The preference for male surgeons was found to be present among male participants (53.1%) and female participants (14.7%) (*p* < 0.001). On the other hand, 49.2% of women preferred female surgeons. The gender preferences of participants in emergency interventions varied considerably. A male surgeon was preferred by a significantly larger proportion of male participants (58.9%) than by any female participants (4.7%; *p* < 0.001). However, 25.9% of women preferred female surgeons. We also investigated surgeon preferences regarding “sensitive” obstetric, genital, and sexual procedures. A total of 68.4% of men favored male surgeons. On the contrary, female surgeons were preferred by 79.2% of the participants (*p* < 0.001). The group more evenly distributed gender preferences for fundamental surgical procedures like abscess drainage. In contrast, female participants (45.2%) exhibited a preference for female surgeons, while male participants (46.2%) favored male surgeons (*p* < 0.001). For significant and major surgical procedures, male participants (56.3%) opted for male surgeons, whereas female participants (27.6%) preferred female surgeons (*p* < 0.001).

## 4. Discussion

Gender disparities in the medical profession have long been a topic of discussion, with surgery being particularly notable for its substantial gender differences. Gender preferences for surgeons differ significantly across a range of surgical visits, including routine non-emergency appointments, emergent cases, delicate surgeries, minor procedures, and major operations, according to this study.

To provide appropriate context for the interpretation of the study results, it is important to consider the demographic characteristics of the study’s participants in relation to the broader population of Saudi Arabia. Saudi Arabia has a total population of approximately 35 million people, with a relatively young demographic profile. About half of the population is above the age of 30 [17]. The country has made significant strides in improving literacy rates, with an estimated 95% of the adult population being literate. Regarding higher education, Saudi Arabia has witnessed substantial advancements in recent years, driven by substantial government investments. More than 70% of young Saudis attend universities. The demographic characteristics of the study’s participants were largely aligned with the general population of Saudi Arabia. The majority of the participants were within the age group of 20 to 40 years, reflecting the country’s youthful demographic composition [18]. Similarly, approximately 80% of the study’s participants had some form of higher education, which is consistent with the national statistics on tertiary education enrollment [19]. This alignment between the study’s sample and the broader population of Saudi Arabia suggests that the study results are likely to be representative of the opinions and perspectives of the majority of the people living in the country. The participants did not exhibit a skewed profile that would limit the generalizability of the findings.

Consistent with previous research [20,21], these results demonstrate that patients frequently have gender-specific preferences regarding their healthcare providers. A large proportion of women favored seeing a female surgeon for non-emergency visits, and male participants favored a male surgeon in this study. This gender-based preference gap may be attributed to patient comfort and the perception that surgeons of the same gender are more empathetic [22]. Prior studies have indicated that patients are more inclined to confide in surgeons of the same gender when it comes to delicate health matters; this inclination is associated with their inclination toward regular visits [23,24]. There was an evident disparity in gender preferences during emergency surgical situations. A minority of female participants expressed the opposite sentiment, with the majority of male participants preferring to have a male surgeon. Societal norms and assumptions may attribute the discrepancy to male surgeons’ attributes of resoluteness and self-assurance in high-pressure situations [25]. Conversely, a considerable number of female respondents exhibited a predilection for female surgeons, potentially attributable to their perception that such professionals possess greater empathy and compassion in critical situations [26]. When it came to cases involving obstetrics, delicate genitals, or sexual concerns, surgeons’ preferences differed significantly by gender. In general, female surgeons were favored by women, whereas men tended to favor male surgeons. Potentially, patients would feel more comfortable and inclined to discuss intimate health concerns with a provider of the same gender, which would foster greater communication and trust [27,28]. The participants’ gender preferences were distributed more equitably during minor surgical procedures like abscess drainage. A marginally smaller but still significant proportion of female participants expressed a preference for female surgeons, whereas a greater number of male participants exhibited a preference for male surgeons. Evaluations of technical expertise, communication approaches, or previous encounters with healthcare professionals of a specific gender may influence patients’ preferences [29,30]. In the context of major surgical procedures, a substantial majority of male respondents indicated a predilection for male surgeons, whereas a notable segment of female respondents expressed no preference for female surgeons. A variety of determinants may impact these preferences. These determinants comprise the provider’s credibility, confidence, and trustworthiness, in addition to the preference for gender concordance in surgical procedures that entail greater invasiveness and protracted recuperation times [31,32]. The results of this study are highly characteristic of Saudi Arabian culture. Similar trends were observed by other researchers, for example, in other Middle Eastern societies with similar cultural backgrounds [33,34]. As indicated in the study of the gender preferences in Oman and Southern Iran, similar results in Saudi Arabia can be explained by the predominant role of particular gender-based norms and values, where men are more applicable for routine surgical procedures, and women feel indifferent regarding emergency and major surgeries [34]. In addition, the study’s results revealed the increased possibility of the female group of patients preferring a female surgeon, especially in routine non-emergency visits and sensitive surgical procedures. Similar results were detected by other researchers in culturally conservative areas, where female patients were reported to be more comfortable with female healthcare professionals for religious and modesty-related reasons [35,36]. Such data confirms that the results of the conducted study align with those of other studies conducted in the Middle East or similar conservative areas, but it also demonstrates that the Saudi Arabian culture also has unique features. For example, increased government investments in the development of higher education and healthcare stipulated enlarged participation of women in these spheres, which was not observed in the past and in other similar conservative societies. This, in turn, can have a profound impact on the public view of female surgeons and will likely prevent the occurrence of particular issues that Saudi women had to face in other Middle Eastern countries [36].

A variety of factors appeared to influence the participants’ preferences regarding the gender of surgeons performing breast cancer and hemorrhoid operations, according to the study’s findings. While both male and female participants indicated a preference for surgeons of the same gender, the rationales behind this variation were not entirely dissimilar. Participants who expressed a preference for female surgeons during breast cancer surgery provided a variety of justifications. These included the perception of female surgeons as courageous, self-assured, patient, seasoned, and proficient, as well as the provision of an environment conducive to candid dialogue due to their gender similarity. Consistent with prior studies, these results indicate that patients might experience greater comfort when discussing delicate health matters with healthcare professionals of the same gender, which, in this instance, would result in a preference for female surgeons [37]. Additionally, the perception of female surgeons as nurturing and empathic may influence the preference for them [37]. Several studies have revealed that when it comes to breast surgery, women prefer female surgeons. Another study discovered that women were more accepting of female surgeons when it came to discussing intimate body-related matters [38]. Similarly, some women may prefer female breast surgeons because they feel more comfortable when a female medical professional examines their breasts. Conversely, people who expressed a preference for male surgeons in breast cancer surgery did so on the grounds that they perceived them to be fearless, self-assured, patient, seasoned, and proficient. Within the Saudi and Muslim communities, there may be a stronger preference for female surgeons when it comes to procedures involving intimate body parts like the breasts [33]. This could be driven by the emphasis on modesty and gender segregation in many Islamic cultures [39]. Patients, especially women, may feel more comfortable discussing health matters related to intimate areas with a same-gender healthcare provider, as this aligns with cultural and religious norms around cross-gender interactions [38]. Additionally, the perception of female surgeons as more nurturing and empathetic may be particularly influential in the Saudi/Muslim context, where these traits are highly valued [40]. Patients may feel that a female surgeon would be better able to provide the compassionate, holistic care that is important in the Islamic medical ethos. Conversely, the preference for male surgeons in some cases may stem from traditional gender role expectations, where men are seen as more authoritative and competent, especially in technical fields like surgery [40]. However, this preference may be counterbalanced by the strong cultural emphasis on modesty and gender segregation when it comes to intimate healthcare. Certain participants in this study cited past adverse encounters with surgeons of the opposite gender as a factor that shaped their gender preference. This implies that individual experiences may have an impact on the formation of patients’ preferences. It is noteworthy that a reduced proportion of respondents cited factors such as enhanced patient examination capabilities and superior professional expertise as justifications for favoring male or female surgeons. This emphasizes the importance that patients place on technical proficiency and meticulousness during the examination procedure, factors that could potentially influence their predilection toward a specific gender.

The present study has certain limitations that warrant careful consideration. The research relied on self-reported preferences, which could potentially contain inaccuracies due to recall bias or social desirability bias. The investigation into the fundamental rationales underpinning the participants’ preferences was insufficient; furthermore, qualitative research could provide more comprehensive insights into the determinants of these preferences. The study’s sample may not be entirely representative of the larger populace, as it includes individuals from a specific geographic region or demographic subset. Moreover, there is a potential lack of external validity and the presence of sampling bias due to the convenience sampling method employed.

## 5. Conclusions

This study demonstrated the complicated nature of the gender preferences of individuals with regard to the choice of the surgeon conducting all sorts of surgical procedures. To evaluate the importance of the results obtained, one must keep in mind that a large number of factors, such as personal experience, cultural peculiarities, comfort of the patient, and perceived knowledge of the situation, can be attributed to gender differences of almost every kind. The fact that numerous variations in preferences depend on the situation underlines the importance of this study. To ensure that the medical care to which patients are exposed is indeed centered around the needs and predispositions of the latter, it is crucial that the corresponding needs should be identified by the healthcare system and staff. Furthermore, this study indicates the existence of gender preferences among the Saudi Arabian population regarding the gender of surgeons who should perform various surgical procedures. Such results are heavily reliant on the existing cultural and social norms of this country, which are predominantly conservative and emphasize the importance of traditional gender roles and modesty in medical settings. Overall, the results of this study indicate the necessity to remain culturally sensitive or even aware in the future related to Saudi Arabia. Such information remains highly beneficial for healthcare professionals as it helps better serve the unique cultural needs and requirements of a particular population and, thus, enhances the provided quality of care.

## Figures and Tables

**Table 1:** Sociodemographic details of the participants.

		N	%
Age	18–25	1067	51.2
	26–35	383	18.4
	36–45	348	16.7
	Above 45	287	13.8
Gender	Male	852	40.9
	Female	1233	59.1
Marital status	Single	1201	57.6
	Married	770	36.9
	Divorced	40	1.9
	Widowed	74	3.5
Nationality	Saudi	1986	95.3
	Non-Saudi	99	4.7
Educational level	General education (primary, intermediate, secondary)	504	24.2
	University	1475	70.7
	Postgraduate	106	5.1
Employment status	I study or work in a healthcare field	560	26.9
	I study or work in a non-healthcare field	905	43.4
	Not working	620	29.7
Area of Residence	Central Region	427	20.5
	Western Region	416	20.0
	Eastern Region	417	20.0
	Southern Region	410	19.7
	Northern Region	415	19.9

**Table 2:** Surgery-related history.

		N	%
Have consulted or undergone treatment by a surgeon or surgeons		1077	51.7
Body region that would be examined by a surgeon of the same sex	Face	491	23.5
	Mouth	399	19.1
	Head region	335	16.1
	Neck region	363	17.4
	Chest region	695	33.3
	Abdomen	623	29.9
	Pelvic region	591	28.3
	Genitals	1073	51.5
	Extremity	283	13.6
	Back	451	21.6
	I did not have any preferences	596	28.6
Reasons for preferring gender of the same gender	Comfortable	679	32.6
	Modesty	69	3.3
	I can talk openly with a surgeon of the same gender	1093	52.4
	Do not believe that a surgeon of the same sex has a greater understanding of the symptoms	127	6.1
	Confusion	117	5.6

**Table 3:** Preferences for breast cancer and hemorrhoid surgery and its relationship with participants’ gender.

			Gender		Total	*p*-Value
			Male	Female		
Surgeon’s gender preferences for breast cancer surgery	No	N	458	641	1099	0.001
		%	53.8%	52.0%	52.7%	
	Yes, female surgeons are always the best	N	186	351	537	
		%	21.8%	28.5%	25.8%	
	Yes, male surgeons are always the best	N	208	241	449	
		%	24.4%	19.5%	21.5%	
Surgeon’s gender preferences for hemorrhoid surgery	No	N	396	589	985	<0.001
		%	46.5%	47.8%	47.2%	
	Yes, female surgeons are always the best	N	74	500	574	
		%	8.7%	40.6%	27.5%	
	Yes, male surgeons are always the best	N	382	144	526	
		%	44.8%	11.7%	25.2%	

**Table 4:** Reasons for choosing surgeon’s gender for breast cancer and hemorrhoid surgery.

Reasons for Choosing a Surgeon		Surgeon’s Gender Preferences for Breast Cancer Surgery			Surgeon’s Gender Preferences for Hemorrhoid Surgery		
		Yes, Female Surgeons Are Always the Best	Yes, Male Surgeons Are Always the Best	Total	Yes, Female Surgeons Are Always the Best	Yes, Male Surgeons Are Always the Best	Total
Fearless	N	52	36	88	73	39	112
	%	9.7%	8.0%	8.9%	12.7%	7.4%	10.2%
Confident	N	77	68	145	54	57	111
	%	14.3%	15.1%	14.7%	9.4%	10.8%	10.1%
Higher level of patience	N	18	29	47	13	22	35
	%	3.4%	6.5%	4.8%	2.3%	4.2%	3.2%
More experienced	N	44	99	143	25	61	86
	%	8.2%	22.0%	14.5%	4.4%	11.6%	7.8%
Competent	N	26	96	122	24	73	97
	%	4.8%	21.4%	12.4%	4.2%	13.9%	8.8%
Can talk openly with a surgeon of the same gender	N	243	58	301	328	211	539
	%	45.3%	12.9%	30.5%	57.1%	40.1%	49.0%
Bad experience with surgeons of opposite gender in the past	N	8	10	18	9	5	14
	%	1.5%	2.2%	1.8%	1.6%	1.0%	1.3%
Superior professional skills	N	20	36	56	16	36	52
	%	3.7%	8.0%	5.7%	2.8%	6.8%	4.7%
Examines patients better	N	49	17	66	32	22	54
	%	9.1%	3.8%	6.7%	5.6%	4.2%	4.9%

**Table 5:** Multivariate analysis testing variables significantly associated with population gender in univariate analysis.

		*p*-Value	aOR	95% C.I.	
Surgeon preferred for regular non-emergency visits to the surgery clinic	Male	<0.001	0.505	0.344	0.742
	Female	<0.001	2.344	1.543	3.560
	No preference	<0.001			
Surgeon preferred for emergency surgical cases	Male	0.837	0.960	0.653	1.413
	Female	0.190	0.683	0.386	1.209
	No preference	0.398			
Surgeon preferred for “sensitive” surgical cases (genital, obstetric, or sexual problems)	Male	<0.001	0.218	0.151	0.314
	Female	<0.001	7.064	5.044	9.895
	No preference	<0.001			
Surgeon preferred in the event that you need a minor surgical procedure (such as draining an abscess)	Male	0.966	0.992	0.672	1.463
	Female	<0.001	2.489	1.628	3.804
	No preference	<0.001			
Surgeon preferred for major surgery (surgical intervention or laparoscopic surgery)	Male	0.164	1.314	0.895	1.928
	Female	0.572	1.165	0.685	1.982
	No preference	0.379			

**Table 6:** Participants’ gender preferences for surgery.

		Gender of Participant		Total	*p*-Value
		Male	Female		
Surgeon preferred for regular non-emergency visits to the surgery clinic	Male	452 (53.1%)	181 (14.7%)	633 (30.4%)	<0.001
	Female	75 (8.8%)	607 (49.2%)	682 (32.7%)	
	No preference	325 (38.1%)	445 (36.1%)	770 (36.9%)	
Surgeon preferred for emergency surgical cases	Male	502 (58.9%)	415 (33.7%)	917 (44%)	<0.001
	Female	40 (4.7%)	319 (25.9%)	359 (17.2%)	
	No preference	310 (36.4%)	499 (40.9%)	809 (38.8%)	
Surgeon preferred for “sensitive” surgical cases (genital, obstetric, or sexual problems)	Male	583 (68.4%)	104 (8.4%)	687 (32.9%)	<0.001
	Female	95 (11.2%)	976 (79.2%)	1071 (51.4%)	
	No preference	174 (20.4%)	153 (12.4%)	327 (15.7%)	
Surgeon preferred in the event that you need a minor surgical procedure (such as draining an abscess)	Male	399 (46.8%)	191 (15.5%)	590 (28.3%)	<0.001
	Female	72 (8.5%)	557 (45.2%)	629 (30.2%)	
	No preference	381 (44.7%)	485 (39.3%)	866 (41.5%)	
Surgeon preferred for major surgery (surgical intervention or laparoscopic surgery)	Male	480 (56.3%)	415 (33.7%)	895 (42.9%)	<0.001
	Female	50 (5.9%)	340 (27.6%)	390 (18.7%)	
	No preference	322 (37.8%)	478 (38.8%)	800 (38.4%)	

## Data Availability

Data are available upon reasonable request by contacting the corresponding author.
